# Carpal Tunnel Syndrome with Wrist Trigger Caused by Hypertrophied Lumbrical Muscle and Tenosynovitis

**DOI:** 10.1155/2015/705237

**Published:** 2015-06-10

**Authors:** Ayuko Shimizu, Masayoshi Ikeda, Yuka Kobayashi, Ikuo Saito, Joji Mochida

**Affiliations:** ^1^Department of Orthopaedics, Tokai University School of Medicine, 143 Shimokasuya, Isehara, Kanagawa 259-0198, Japan; ^2^Department of Orthopaedic Surgery, Shonan Central Hospital, 1-3-43 Hatori, Fujisawa, Kanagawa 251-0056, Japan

## Abstract

We present a case of carpal tunnel syndrome involving wrist trigger caused by a hypertrophied lumbrical muscle with flexor synovitis. The case was a 40-year-old male heavy manual worker complaining of numbness and pain in the median nerve area. On active flexion of the fingers, snapping was observed at the carpal area, and forceful full grip was impossible. Tinel's sign was positive and an electromyographic study revealed conduction disturbance of the median nerve at the carpal tunnel. Magnetic resonance imaging revealed edematous lumbrical muscle with synovial proliferation around the flexor tendons. Open carpal tunnel release was performed under local anesthesia. Synovial proliferation of the flexor tendons was found and when flexing the index and middle fingers, the lumbrical muscle was drawn into the carpal tunnel with a triggering phenomenon. After releasing the carpal tunnel, the triggering phenomenon and painful numbness improved.

## 1. Introduction

Carpal tunnel syndrome (CTS) is one of the most commonly encountered compression neuropathies, and most cases are idiopathic [1, 2]. We report the case of a patient who had CTS with triggering wrist caused by hypertrophy of a lumbrical muscle and tenosynovitis due to overuse from heavy labor. The patient showed a characteristic symptom that the numbness became worse with a snapping phenomenon at the carpal area when flexing the fingers. Magnetic resonance imaging (MRI) and an electromyographic study (EMG) confirmed a mechanical disturbance against the median nerve.

## 2. Case Report

**Figure 1:**
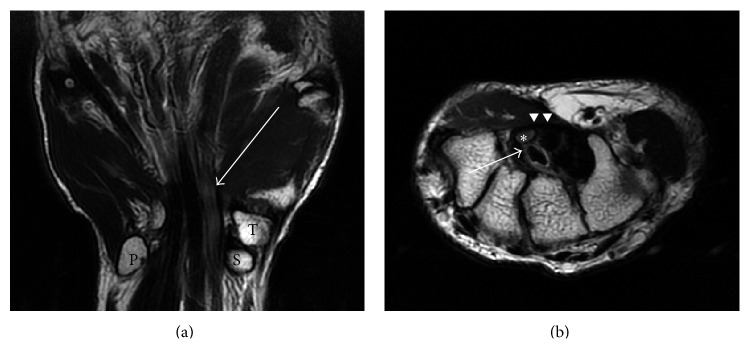
Magnetic resonance imaging of the wrist. (a) T2-weighted coronal image. Edematous lumbrical muscle with synovial proliferation (white arrow) was seen around the flexor tendons. T: trapezium, S: scaphoid, P: pisiform. (b) T2-weighted axial image. The median nerve (*∗*) was compressed between the transverse carpal ligament (white arrow heads) and the edematous synovial tissue (white arrow) around the flexor tendon.

A 40-year-old male was referred to our institution one month after he felt numbness and pain in his right hand. He engaged in hard manual labor for the past five years, which involved loading/unloading of about 500 parcels of 10 kg weight every day. Physical examinations showed hyperesthesia and pain in the median nerve area. On active flexion of the index and middle fingers, a convex shape was observed at the carpal area with snapping, which exaggerated the symptoms. Evident thenar muscle atrophy was not observed. Tinel's sign, Phalen's test, and fist test results were positive. Plain radiographs of the wrist showed no abnormalities. Magnetic resonance imaging revealed edematous lumbrical muscle with synovial proliferation around the flexor tendons (Figure 1(a)). The median nerve was compressed between the transverse carpal ligament and the edematous muscle and synovial tissue (Figure 1(b)). Motor nerve conduction studies of the median nerve revealed evident prolonged distal latency. Sensory nerve conduction studies showed prolonged latency and delayed conduction velocity. Needle EMG (electromyography) showed no denervation potentials at rest. On voluntary contraction, there was no positive sharp wave but a few polyphasic motor units were detected. These findings were compatible to moderate neurophysiological CTS grading according to Stevens' classification [3].

**Figure 2:**
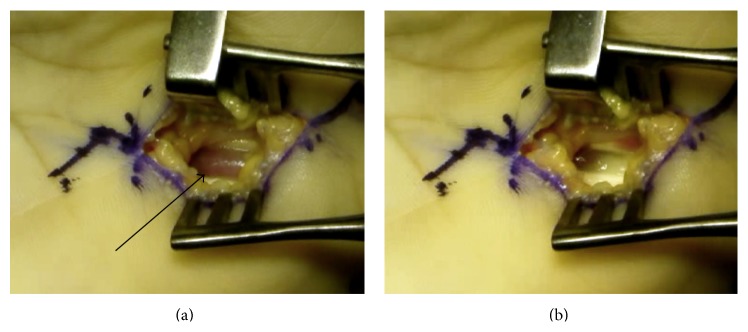
Intraoperative findings. (a) On finger flexion, the lumbrical muscle belly (black arrow) moves proximally into the carpal tunnel with snapping and occupies the tunnel. (b) On finger extension, the lumbrical muscle belly moves distally and is swept out of the tunnel.

**Figure 3:**
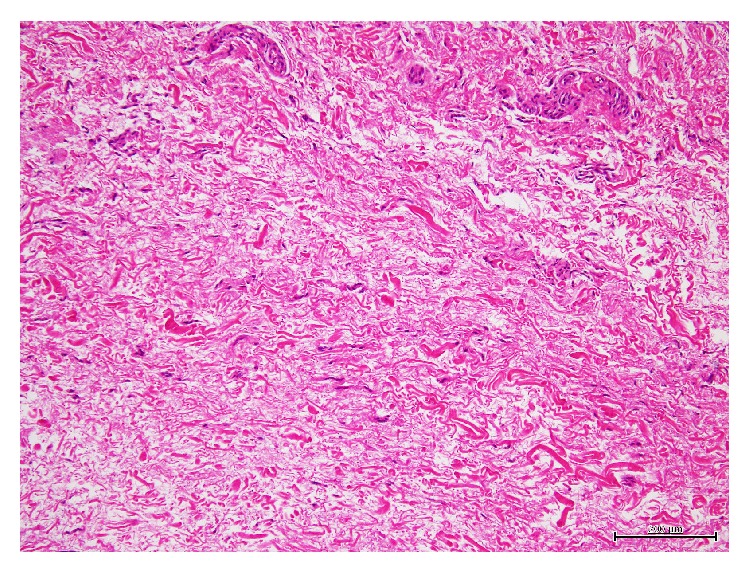
Histopathological finding of the resected synovium. The resected synovium mainly consisted of dense reactive proliferation of fibrous tissue with scattered inflammatory cells (hematoxylin and eosin stain; original magnification, ×40).

Thus, open carpal tunnel release was performed under local anesthesia. Synovial proliferation of the flexor tendons was found. The median nerve showed no evident abnormality. On flexing the index and middle fingers, the lumbrical muscle was drawn into the carpal tunnel with a triggering phenomenon at the distal edge of the flexor retinaculum, and the muscle belly occupied the tunnel and compressed the flexor tendons with the medial nerve (Figure 2(a)). On extending the fingers, the lumbrical muscle moved distally and disappeared from the tunnel (Figure 2(b)). After completely releasing the transverse carpal ligament proximally and distally, partial synovectomy around the flexor tendons, it was confirmed that the wrist triggering phenomenon did not happen. Then, lumbrical muscle belly was not resected. Histopathologically, the resected synovium mainly consisted of dense reactive inflammatory proliferation of fibrous tissue (Figure 3).

The triggering phenomenon at the wrist and painful numbness disappeared immediately after the operation. The Disabilities of the Arm, Shoulder, and Hand score improved postoperatively (Disability/Symptom, 69.2, work, 100 before operation; Disability/Symptom, 1.67, work, 0 at three months after the operation). There has been no recurrence of the symptoms two years after the operation.

## 3. Discussion

Lumbrical muscles originate from the flexor digitorum profundus tendon, and the muscle belly moves proximally on finger flexion. Several authors reported that the lumbrical muscle intrusion was a potential cause of CTS [4–7]. There have been a few reports of CTS associated with lumbricals. There are various underlying causes of this condition, such as anomaly of the muscle [8, 9], tumor-related conditions [10], and hypertrophy from overuse [11, 12]. In the present case, although there was no evident lumbrical anomaly, it is supposed that repetitive long-standing overuse induced the hypertrophy of the muscle with tenosynovitis, which introduced the triggering wrist phenomenon and elevated the internal pressure of the tunnel. CTS associated with occupations in which overuse induces lumbrical hypertrophy are characterized by the following aspects: most patients being young or middle-aged males, single-side involvement, deteriorated symptoms on finger flexion, positive fist test results, triggering of the wrist, and resistance to conservative treatment [6, 7, 11–13]. In addition to the above characteristics, MRI showed signal changes along the flexor tendon and lumbrical muscle adjacent to the median nerve that suggested edematous synovial proliferation or synovitis. Although the EMG do not reveal severe neuropathic changes of the abductor pollicis brevis, mechanical stress due to lumbrical muscle and proliferated synovia induces severe irritable neurogenic pain and the patient cannot continue manual labor.

Operative treatment is preferred for CTS that is resistant to conservative treatment. Carpal tunnel release with synovectomy is usually performed and synovial proliferation is often seen, but in some cases, resection of the lumbrical muscle is performed [11, 12]. The prognosis is good after operative treatment, because it is usually performed at the early stage when the irreversible pathological changes do not occur in the nerve.
